# Epinephrine in Anaphylaxis: Preclinical Study of Pharmacokinetics after Sublingual Administration of Taste-Masked Tablets for Potential Pediatric Use

**DOI:** 10.3390/pharmaceutics10010024

**Published:** 2018-02-11

**Authors:** Ousama Rachid, Mutasem Rawas-Qalaji, Keith J. Simons

**Affiliations:** 1 College of Pharmacy, Qatar University, P.O. Box 2713 Doha, Qatar; 2 College of Pharmacy, Health Professions Division, Nova Southeastern University, Fort Lauderdale, FL 33328, USA; 3 College of Pharmacy, Faculty of Health Sciences, University of Manitoba, Winnipeg, MB R3E 0T5, Canada; Keith.Simons@umanitoba.ca

**Keywords:** bioavailability, bioequivalence, intramuscular, auto-injector, sublingual delivery, rapidly-disintegrating, tablets, allergy, anaphylaxis, adrenaline, epinephrine

## Abstract

Epinephrine is a life-saving treatment in anaphylaxis. In community settings, a first-aid dose of epinephrine is injected from an auto-injector (EAI). Needle phobia highly contributes to EAI underuse, leading to fatalities—especially in children. A novel rapidly-disintegrating sublingual tablet (RDST) of epinephrine was developed in our laboratory as a potential alternative dosage form. The aim of this study was to evaluate the sublingual bioavailability of epinephrine 30 mg as a potential pediatric dose incorporated in our novel taste-masked RDST in comparison with intramuscular (IM) epinephrine 0.15 mg from EAI, the recommended and only available dosage form for children in community settings. We studied the rate and extent of epinephrine absorption in our validated rabbit model (*n* = 5) using a cross-over design. The positive control was IM epinephrine 0.15 mg from an EpiPen Jr^®^. The negative control was a placebo RDST. Tablets were placed under the tongue for 2 min. Blood samples were collected at frequent intervals and epinephrine concentrations were measured using HPLC with electrochemical detection. The mean ± SEM maximum plasma concentration (*C*_max_) of 16.7 ± 1.9 ng/mL at peak time (*T*_max_) of 21 min after sublingual epinephrine 30 mg did not differ significantly (*p* > 0.05) from the *C*_max_ of 18.8 ± 1.9 ng/mL at a *T*_max_ of 36 min after IM epinephrine 0.15 mg. The *C*_max_ of both doses was significantly higher than the *C*_max_ of 7.5 ± 1.7 ng/mL of endogenous epinephrine after placebo. These taste-masked RDSTs containing a 30 mg dose of epinephrine have the potential to be used as an easy-to-carry, palatable, non-invasive treatment for anaphylactic episodes for children in community settings.

## 1. Introduction

Prompt injection of epinephrine in the mid-outer thigh (vastus lateralis muscle) using an auto-injector is the recommended first-aid treatment of anaphylaxis in community settings [1]. Many patients at risk of anaphylaxis in the community fail to carry their epinephrine auto-injectors consistently, due to their bulky shape and large size [2]. When anaphylaxis occurs, many patients and caregivers who have an epinephrine auto-injector available were reported to delay injecting epinephrine because of their fear of needles [3,4,5]. Other issues include a short shelf-life and availability of only two fixed doses (0.15 and 0.3 mg) for patients ranging in weight from <5 kg to >125 kg [2]. There is an increasingly challenging availability and affordability issue of epinephrine autoinjectors worldwide, with pharmacy acquisition costs in North America ranging from $170 to $430 US dollars per pack [6]. This is compounded by the need for multiple devices to be placed in various locations as part of the user’s preparedness plan, such as home, work, school, and during traveling; and the need to replace expired devices almost every year. Manual techniques of removing and administering second or third epinephrine doses from used devices and filling or prefilling injections from epinephrine ampules have been suggested to overcome the high cost of autoinjectors; however, the accuracy, safety, and practicality of these techniques are questionable [7,8,9,10].

Rapidly-disintegrating sublingual tablets (RDSTs) of epinephrine have been developed as a potential non-invasive alternative epinephrine dosage form for the treatment of anaphylaxis in community settings. The highly vascular sublingual mucosa facilitates rapid drug absorption into the venous circulation through the sublingual veins [11]. Epinephrine bitartrate, a low molecular weight hydrophilic compound, is absorbed by passive diffusion driven by a concentration gradient. The high drug concentration in the sublingual space drives the drug through the mucosal epithelium into the interstitial fluid, to then be absorbed by the venous circulation [11].

In our initial preclinical studies, a dose-escalation study (10, 20, and 40 mg) was performed to determine the sublingual epinephrine dose that is bioequivalent to the intramuscular adult dose of epinephrine 0.3 mg [11]. Results showed that the administration of a first-generation RDST of epinephrine 40 mg formulation resulted in plasma epinephrine concentrations similar to those achieved after the administration of an adult dose of epinephrine 0.3 mg by intramuscular injection [11].

Later, these RDSTs were found to have a shelf-life of up to 7 years [12]. The rate of complete epinephrine dissolution was also optimized by altering excipient proportions to reach ≤60 s following fast tablet disintegration in ≤30 s [13,14,15,16]. The intrinsic bitter taste of epinephrine in the sublingual tablets was then masked by adding a taste masking excipient (citric acid), in addition to other excipients [17], since the bitter taste can be a potential barrier for patients’ compliance, particularly for pediatric use. The absorption of epinephrine 40 mg from these tasted-masked sublingual tablet formulations was reevaluated again in animal model [18].

Combining the findings from the dose-escalation and taste-masking studies, we hypothesized that a taste-masked RDST formulation with a lower epinephrine dose of 30 mg would have the potential as a child dose for the treatment of anaphylaxis in a pediatric population. To our knowledge, this is the first pre-clinical study of a potential pediatric sublingual dose of epinephrine for the treatment of anaphylaxis.

The assessment of new pediatric dosage forms, new dose regimens, or new routes of administration of certain drugs and biologics was made mandatory by the FDA as per the Pediatric Research Equity Act (PREA) in 2003 [19]. According to the act, adequate pharmacokinetic data supporting dosing and administration for each pediatric subpopulation—permitting acceptable extrapolation between age groups—is a required part of the application process. The pediatric product should be a user-friendly easy-to-swallow or dissolvable dosage form with acceptable palatability. The product should also provide adequate bioavailability and be stable over a range of conditions. Taste-masking of formulations must take into consideration the effect of sweetening and/or flavoring agents on the pharmacokinetic profiles of medications being masked for their unpleasant taste.

Therefore, our objective in this preclinical study was to evaluate the pharmacokinetic profile of an epinephrine 30 mg dose from taste-masked rapidly-disintegrating sublingual tablets as a potential pediatric dose in comparison to epinephrine 0.15 mg intramuscular injection from EpiPen Jr^®^, the only available pediatric dose in epinephrine auto-injectors.

## 2. Materials and Methods

### 2.1. Manufacturing of Taste-Masked Rapidly-Disintegrating Sublingual Tablets (RDSTs) of Epinephrine

The composition of the formulation used to manufacture taste-masked RDSTs is shown in Table 1. Epinephrine bitartrate 54.58 mg, equivalent to 30 mg of epinephrine, was used in the preparation of epinephrine RDST (Epi 30). The ratio of total microcrystalline cellulose (both PH-301 and PH-M-06) to low-substituted hydroxypropyl cellulose was kept at 9:1 in the placebo and Epi 30 RDST formulations. This pre-determined ratio enabled optimal disintegration times, as reported previously [14,15]. Magnesium stearate was used as a lubricant and kept at 2% in a total tablet weight of 200 mg.

A 13/32 (0.4062 inch) die with flat face upper and lower punches (Natoli Engineering Company, Inc., St. Charles, MO, USA) was used to manufacture RDSTs by direct compression at a preselected range of compression forces (CFs, 18.5–23.25 kN) using a Manesty-F3 single-punch tablet press machine (Liverpool, UK) [15]. A dial caliper (Hempe Manufacturing Co., Inc., New Berlin, WI, USA) was used to measure the dimensions, diameter, and thickness of the compressed tablets.

### 2.2. Quality Control Testing of Taste-Masked Rapidly-Disintegrating Sublingual Tablets (RDSTs) of Epinephrine

Tablet weight variation and drug content uniformity were measured following the USP methods and criteria [20]. To determine tablet weight variation, an analytical balance (Mettler-Toledo Inc., Columbus, OH, USA) was used to individually weigh 10 out of 30 randomly selected tablets. Drug content was analyzed using a high-performance liquid chromatography (HPLC) system with ultraviolet (UV) detection at 280 nm (Waters Corp., Milford, MA, USA). An acceptance value (AV) of 15.0 was used, according to the harmonized USP method.

A hardness tester (Erweka, Heusenstamm, Germany) was used to measure the breaking force of six tablets selected randomly from each formulation batch. A friability tester (Pharma Test Apparatebau GmbH, Hainburg, Germany) was used to determine the friability according to the USP guidelines to measure the friability of compressed, uncoated tablets [21]. Briefly, the drum of the friability tester, containing a random sample of whole and dedusted tablets corresponding to 6.5 g, was rotated 100 times and tablets were removed, dedusted, and accurately reweighed. A friability value of ≤1.0% weight loss was considered acceptable.

Due to the absence of an appropriate dissolution apparatus and method that simulates the physiological conditions in the sublingual cavity, a validated novel in vitro method was followed to test the dissolution of epinephrine from RDSTs using a custom-made dissolution apparatus constructed in our laboratory [13]. The dissolution medium of 2 mL of distilled water was added into a donor glass funnel that is 15 mL in volume capacity, into which a tablet was placed to disintegrate and dissolve for 120 s without any agitation or motion. Using a vacuum pump, further drug dissolution was terminated by withdrawing the total volume of the dissolution medium into the collection tube passing through a 0.45 µm filter membrane. The dissolved drug content in the filtrate was measured by HPLC with UV detection (Waters Corp.) according to the official USP assay for Epinephrine Injection [22]. The percentage of drug dissolved (DD%) was calculated by dividing the drug content (mg) in the filtrates of six individual RDSTs by the content uniformity value of the tablet formulation batch.

### 2.3. Animal Study Design

A randomized three-arm cross-over placebo-controlled study was performed in New Zealand female white rabbits (*n* = 5), an epinephrine-tolerant species (mean weight ± SD = 3.6 ± 0.1 kg), using a previously reported protocol [11,18]. The studies were performed in three different study days (one treatment/arm/day) at least 4 weeks apart, as a wash-out period and to replenish blood volume. The rate and extent of epinephrine absorption from Epi 30 sublingual tablets were investigated in comparison to epinephrine absorption following 0.15 mg intramuscular injection in the mid-outer thigh using EpiPen Jr^®^ as a positive control. In-date EpiPens Jr^®^ 0.15 mg (Mylan Specialty L.P, Basking Ridge, NJ, USA) were purchased from the University of Manitoba pharmacy. Placebo RDSTs containing identical excipients composition and ratios of Epi 30 were used as the negative control. 

The project was approved by the University of Manitoba Protocol Management and Review Committee. The guidelines published by the Canadian Council on Animal Care were followed throughout.

On each study day, an indwelling catheter was inserted into an ear artery >30 min before dosing. Blood samples of 2 mL per sample were withdrawn immediately before dosing to obtain baseline readings (endogenous epinephrine), and 5, 10, 15, 20, 30, 40, and 60 min after dosing for the measurement of plasma epinephrine concentrations.

The technique of administering sublingual tablets into the rabbit’s mouth was modified from the one previously reported [11]. Briefly, the rabbit mouth was opened with the aid of a speculum, after which the tablet was placed carefully under the tongue with the aid of forceps and was kept undisturbed for 2 min [18]. Then, the tablet residues were removed from the rabbit mouth by washing with 40–50 mL distilled water to terminate any further epinephrine absorption.

### 2.4. Measurement of Plasma Epinephrine Concentrations

Blood samples were collected in a BD Vacutainer^®^ PPTM Plasma Preparation Tubes, refrigerated within 1 h of sampling, and centrifuged at 4 °C. Plasma samples were frozen at −20 °C. Before analysis, plasma samples were thawed at room temperature, and epinephrine was extracted by a solid-phase extraction (SPE) process, with an efficiency of 70–80% [11], which was improved to 80–90% by optimizing the SPE conditions [23]. An aqueous solution containing 0.1 M perchloric acid (Fisher, Fair Lawn, NJ, USA) and 0.1 mM sodium metabisulfite (Sigma, St. Louis, MO, USA) to maintain the stability of epinephrine, was used for the preparation of all epinephrine stock solutions and subsequent dilutions, and for the desorption of epinephrine from alumina during epinephrine extraction from plasma samples.

A 0.5 mL volume of plasma was added to alumina, along with 50 μL of 0.1 mM sodium metabisulfite (Sigma, St. Louis, MO, USA), 400 μL of tris buffer, and precalculated concentrations of dihydroxybenzylamine (DHBA) (Sigma, St. Louis, MO, USA) as an internal standard, corresponding to the concentrations used in the calibration curve. The mixture was vortexed for 15 min to extract epinephrine and DHBA from the plasma samples, and then washed two times with distilled water to remove any plasma components and buffer. A 100 μL volume of 0.1 M perchloric acid and 0.1 mM sodium metabisulfite (1:1) solution was added, and then vortexed for 5 min to elute epinephrine and DHBA from alumina. After centrifugation, the supernatant solution was transferred into vials for injection into the HPLC system.

Epinephrine was measured using reverse-phase high performance liquid chromatography (Waters Corp.) with electrochemical detection. The potential of the glassy carbon working electrode was set at +600 mV versus ISAAC reference electrode and the detector sensitivity was set at 10 nA. All chromatography was performed on a reversed-phase Nova-Pak^®^ C18 column, 3.9 mm × 150 mm, 60 nominal pore size, 4 μm spherical particles (Waters Corp., Milford, MA, USA). The injection volume was 20 μL.

The mobile phase was composed of buffer:methanol at a ratio of 95:5 (by volume), according to recommendations from Waters^®^. The buffer used was 50 mM sodium acetate (Fisher, Fair Lawn, NJ, USA), 20 mM citric acid (Fisher, Fair Lawn, NJ, USA), mixed with 3.75 mM 1-heptanesulfonic acid sodium salt (Sigma, St. Louis, MO, USA), 0.134 mM EDTA disodium salt dihydrate (Sigma, St. Louis, MO, USA), and 1 mM dibutylamine (Fisher, Fair Lawn, NJ, USA), and filtered using 22 μm nylon membrane filters (Whatman, Whatman International Ltd., Maidstone, UK). The flow rate was set at 1.0 mL/min. Under these conditions, epinephrine and DHBA eluted at 1.9 and 2.5 min, respectively.

Two stock solutions of epinephrine (25 and 250 ng/mL) were prepared using (−)-epinephrine (+) bitartrate (Sigma, St. Louis, MO, USA) and then used to prepare two sets of epinephrine standards ranging from 0.1 to 1.0 ng/mL and from 1.0 to 10.0 ng/mL spiked in anticoagulated rabbit plasma. A 40 μL volume of DHBA 5 ng/mL (0.2 ng) and a 50 μL volume of DHBA 50 ng/mL (2.5 ng) were used with the low and high range calibration curves, respectively. The low-range calibration curve was linear (*R*^2^ of >0.95) over the range 0.1–1 ng/mL (CV%, 0.4–0.1%). The high-range calibration curve was linear (*R*^2^ of >0.99) over the range of 1–10 ng/mL (CV%, 0.1%).

The extraction recovery from plasma was 80–90%. The CV% of the system reproducibility in solution at 1.0 ng/mL (*n* = 5) was 0.25%. The detection limit was 5 pg with a CV% of 28.8% (*n* = 2).

### 2.5. Data Analysis

Mean ± SEM maximum plasma epinephrine concentration (*C*_max_), the time at which *C*_max_ was achieved (*T*_max_), and the area under the plasma epinephrine concentration versus time curve (AUC_0–1 h_) were calculated from the epinephrine versus time plots of each individual rabbit using WinNonlin 5.3 (Pharsight, Mountain View, CA, USA). Values were compared using ANOVA and Tukey–Kramer tests (NCSS Statistical Analysis Software). Differences were considered significant at *p* < 0.05.

## 3. Results

The manufactured taste-masked RDSTs resulted in acceptable tablet weight variation, drug content uniformity, breaking force, and friability. Epinephrine from the manufactured RDSTs was dissolved completely within 2 min. Table 2 summarizes the results of the quality control tests of taste-masked RDSTs.

**Figure 1:**
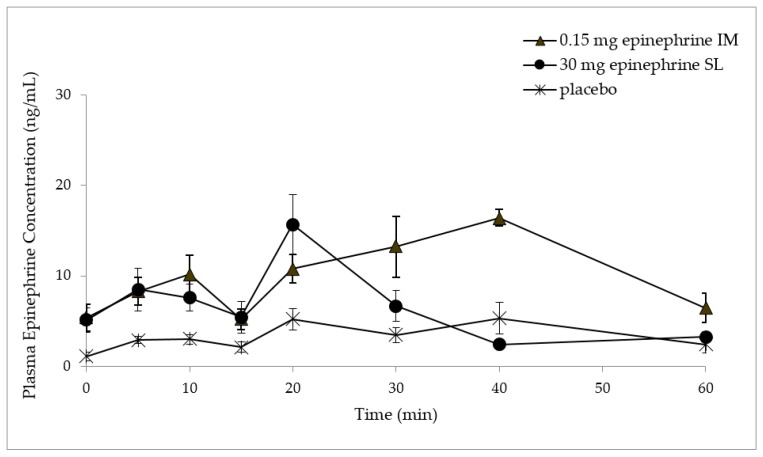
Plasma epinephrine concentration (mean ± SEM) versus time plots following the administration of epinephrine 0.15 mg by intramuscular injection, epinephrine 30 mg sublingually, and placebo sublingually.

The plasma concentration of epinephrine versus time profiles following the administration of placebo and epinephrine 30 mg sublingual tablets, and epinephrine 0.15 mg by intramuscular injection are presented in Figure 1 as means ± SEM. *C*_baseline_, *T*_max_, *C*_max_, and AUC_0–1 h_ values are presented in Table 3 as means ± SEM. The *C*_baseline_ obtained following catheterization of rabbits and just before dosage forms’ administration were not significantly different between the three different treatment arms (*p* ≥ 0.05). The *C*_max_ and *T*_max_ values did not differ significantly after the administration of epinephrine 30 mg by sublingual tablets or epinephrine 0.15 mg by intramuscular injection (*p* ≥ 0.05). However, the AUC_0–1 h_ obtained after the sublingual administration of epinephrine 30 mg was significantly lower than those obtained after the intramuscular injection of epinephrine 0.15 mg (*p* ≤ 0.05). The *C*_max_ and AUC_0–1 h_ following the administration of epinephrine 30 mg sublingual tablets or epinephrine 0.15 mg by intramuscular injection were significantly higher (*p* < 0.05) than the *C*_max_ and AUC_0–1 h_ following the administration of placebo sublingual tablets reflecting the endogenous epinephrine levels.

## 4. Discussion

Visits to emergency departments due to anaphylaxis have been increasing over the years, with the highest number of visits being among children [24]. The management of anaphylaxis includes the administration of epinephrine as the drug of choice. For the first-aid treatment of anaphylaxis, autoinjectors delivering 0.15 mg of epinephrine are prescribed for children, but they are underused for a number of reasons—one of which is needle phobia. Physical injuries resulting from inadvertent and incorrect administration leading to lacerations and embedded needles caused by epinephrine autoinjector use in children have been reported [6].

Potential alternative routes to epinephrine intramuscular administration have been proposed, including inhalational route, in an effort to provide a user-friendly dosage form of epinephrine [25,26]. However, inhalers for asthma as well as autoinjectors for anaphylaxis were associated with misuse, which indicates the need for the extensive training of all caregivers [27]. In our laboratory, a rapidly-disintegrating tablet formulation of epinephrine for sublingual administration has been extensively studied [11,12,13,14,15,16,17,18,28,29]. A rabbit model was utilized for the evaluation of sublingual absorption and pharmacokinetic modeling, which has been shown to be used for many other drugs [30,31,32,33]. The challenges associated with the intramuscular administration of epinephrine have been effectively considered and overcome through the development of a rapidly-disintegrating sublingual tablet formulation of epinephrine. Compared to the intramuscular route, the sublingual route is accessible, convenient for self-administration, and has long been used for self-treatment in other medical emergencies, such as the initial treatment of angina using user-friendly sublingual nitroglycerine tablets. The design and development of taste-masked RDSTs of epinephrine enabled the application of human factor analysis, taking real-life scenarios of human use into consideration. The RDSTs are small in size, and can be easily and conveniently carried anytime and anywhere. These taste-masked RDSTs may be formulated to contain several dose ranges to accommodate the general population on a mg/kg basis.

There is a growing demand for pediatric regulatory requirement to ensure the safety and efficacy of medications in the pediatric population [34,35,36]. Masking the bitter taste of medications is becoming one of the major considerations in the development of a pediatric formulation to enhance administration acceptability by children. The sour taste, provided by the flavoring agent citric acid, is one of the recognized and well-accepted tastes by children and is commonly used in children’s drinks, food, and medications [34]. Epinephrine’s inherent bitter taste in the manufactured tablets was effectively masked by the addition of citric acid as we showed previously in our taste-masking studies using an electronic tongue [17]. However, taste-masking should not compromise the pharmacokinetics of the active pharmaceutical ingredient in the developed pediatric formulation.

In this study, it has been shown that the addition of citric acid as a taste-masking and flavoring agent did not affect the dissolution, absorption, or pharmacokinetics of a potential epinephrine pediatric dose from these developed taste-masked RDSTs, and were similar to the dissolution of our previously published data of non-taste-masked RDSTs [11,14]. Epinephrine 30 mg was completely released from the taste-masked sublingual tablets and dissolved in 2 min, which shows that the addition of citric acid to the tablet formulation did not slow down epinephrine dissolution—a critical and limiting step for epinephrine absorption.

In comparison to the intramuscularly administered pediatric dose of EpiPen Jr^®^ 0.15 mg, the sublingually-administered epinephrine 30 mg was rapidly absorbed following its complete dissolution through sublingual mucosa, resulting in a similar maximum concentration (*C*_max_) at a similar *T*_max_, which are clinically significant parameters for the treatment of anaphylaxis, demonstrating that the addition of citric acid to the tablet formulation did not affect the extent and rate of epinephrine absorption, respectively (Table 3). Despite of the lack of a significant difference in the *T*_max_ due to the small sample size and sublingual variability, the shorter *T*_max_ after sublingual administration of epinephrine compared to *T*_max_ after intramuscular administration is in agreement with results from our previous work [11,18,29]. This can be attributed to the thin mucosa and the abundant blood supply in the sublingual area, facilitating the rapid absorption of epinephrine by passive diffusion across the epithelium into the interstitial fluid.

The administration of epinephrine resulted in two peaks at 5 min and 20 min after the administration of epinephrine 30 mg taste-masked RDSTs compared to two peaks at 10 min and 40 min after IM injection of EpiPen Jr^®^ 0.15 mg (Figure 1).

Similar to what we have reported previously in both animal model and humans [11,18], epinephrine administration through all studied routes of administration resulted in an intermittent pattern of absorption as reflected in two or more peaks of epinephrine in the collected plasma over the duration of the study. Initially, the rapid absorption of epinephrine resulted in the first peak, which led to vasoconstriction at the administration site (i.e., sublingual mucosa or skeletal muscle). The first absorbed portion of epinephrine, consequently leading to vasoconstriction, resulted in a reduction of epinephrine absorption that was temporary due to blood circulation sink condition. However, the remaining higher portion of epinephrine dose continued to accumulate at the site of absorption and interstitial space. Therefore, the subsequent vasodilation due to the elimination of epinephrine resulted in a second phase of epinephrine absorption from the site of absorption, leading to a second, often higher, peak in the systemic circulation due to the accumulation of a larger amount of epinephrine compared to the one resulted in the first peak.

Achieving high epinephrine plasma peaks as rapidly as possible is a clinical necessity to reverse the life-threatening signs and symptoms of anaphylaxis. Epinephrine administered sublingually in a relatively high dose compared with the doses administered intramuscularly was found necessary to create the high concentration gradient that drives its diffusion through the sublingual mucosa according to Fick’s law. Despite the similar magnitude of *C*_max_ resulting after the administration of Epi 30 and EpiPen Jr^®^ 0.15 mg, their AUC_0–1 h_ were significantly different. Paradoxically and despite its half dose, EpiPen Jr^®^ 0.15 mg resulted in similar, but slightly higher AUC_0–1 h_ (654 ng/mL/min) than that achieved after EpiPen^®^ 0.3 mg (592 ng/mL/min) reported previously [18]. It has been shown that further epinephrine absorption from EpiPen Jr^®^ beyond 1 h might occur [37], but it would be clinically insignificant during anaphylaxis episodes when the initial epinephrine peaks in the first hour are critical for life-saving. The AUC_0–1 h_ achieved after the sublingual administration of epinephrine 30 mg in this study is about half the AUC_0–1 h_ achieved after the intramuscular administration of epinephrine 0.3 mg using EpiPens^®^ from previously reported data [18]. The ratio *F.Dose/AUC*_0−1 h_ after sublingual administration of epinephrine 30 mg was 81 F L/min and the ratio calculated after sublingual administration of epinephrine 40 mg from data reported previously [18] was 59 F L/min. Assuming similar clearances, the bioavailability, F, of the 40 mg dose is higher than that of the 30 mg dose, reflecting a higher driving force of sublingual absorption with higher epinephrine doses.

A narrower dose-ranging study of epinephrine in RDSTs should be performed to determine the equivalent sublingual dose to the 0.15 mg intramuscular dose. Epinephrine microcrystals were developed in our laboratory, enhancing epinephrine absorption from RDSTs, which facilitated dose reduction [29]. Tablet dosage form and size suitability for pediatric population have been reviewed, showing positive acceptability of tablet dosage form by age groups ranging from 1 month to 18 years; and mini tablets by age groups ranging from newborns to 5 years [38,39,40]. A range of tablet and mini-tablet sizes can be manufactured that would enable proper administration to meet the needs of different pediatric age groups. In these pediatric age groups, sublingual administration techniques are yet to be evaluated for innovative approaches that are user-friendly, misuse resistant, and economical.

## 5. Conclusions

Taste-masked rapidly-disintegrating sublingual tablets containing epinephrine 30 mg resulted in comparable pharmacokinetic profiles with similar maximum concentrations, but different area under the curve, compared to intramuscular epinephrine 0.15 mg from EpiPen Jr^®^. Further pharmacokinetic studies are needed to determine dose equivalency in preclinical animal models. RDSTs of epinephrine might eventually be useful as an easy-to-carry, palatable, non-invasive treatment for anaphylactic episodes in community settings.

## Figures and Tables

**Table 1:** The type and amounts of ingredients used in the taste-masked rapidly-disintegrating sublingual tablet formulations ^1^.

Ingredient (mg) ^2^	Formulations	
	Placebo	Epi 30
Epinephrine bitartrate	0	54.58
Microcrystalline cellulose (Ceolus^®^ PH-301)	123.00	80.86
Microcrystalline cellulose (Ceolus^®^ PH-M-06)	20.50	13.48
Mannitol (Ludiflash)	34.10	34.10
Citric acid	2.50	2.50
Low-substituted hydroxypropyl cellulose (LH11)	15.90	10.48
Magnesium stearate	4.00	4.00

^1^ Tablet weight was maintained at 200 mg; ^2^ Ratio of total microcrystalline cellulose (Ceolus^®^ PH-301 and Ceolus^®^ PH-M-06) to low-substituted hydroxypropyl cellulose (LH11) was kept at 9:1 in both formulations.

**Table 2:** Mean ± SD diameter, weight variation (WV), content uniformity (CU), breaking force (BF), friability (F), and drug dissolution (DD) for the taste-masked rapidly-disintegrating sublingual tablet formulations.

Characteristics	Formulations	
	Placebo	Epi 30
Diameter (mm)	9.98 ± 0.01	9.98 ± 0.01
WV (mg), (AV) ^a^	202 ± 2.58 (3.1)	211 ± 2.85 (6.47)
CU (%), (AV) ^a^	N/A	102 ± 4.77 (10.94)
BF (kgf)	2.53 ± 0.02	2.50 ± 0.01
F (%)	0.1	0.7
DD (%) ^b^	N/A	102.97 ± 8.28

^a^ AV, USP acceptance value (values ≤15.00 were considered acceptable according to USP L1 limit); ^b^ DD (%), Percentage of drug dissolved in the first 120 s.

**Table 3:** The pharmacokinetic parameters of epinephrine following the sublingual administration of epinephrine 30 mg and placebo tablets and epinephrine 0.15 mg by intramuscular injection in the thigh.

Mean ± SEM *	Placebo Sublingual Tablets (Endogenous Epinephrine)	Epinephrine Sublingual Tablets (Epi 30)	EpiPens Jr^®^
Epinephrine dose (mg)	0	30	0.15
*C*_baseline_ (ng/mL)	1.1 ± 0.5	5.1 ± 1.4	5.4 ± 1.5
*C*_max_ (ng/mL)	7.5 ± 1.7 ^†^	16.7 ± 1.9	18.8 ± 1.9
*T*_max_ (min) ^††^	33.3 ± 7.2	21.0 ± 2.5	36.0 ± 2.5
AUC_0–1 h_ (ng/mL/min)	220.1 ± 31.8 ^†^	372.3 ± 21.7 ^†^	654.2 ± 39.6

*C*_baseline_: baseline plasma concentration reflecting endogenous epinephrine; *C*_max_: maximum plasma concentration (mean ± SEM of individual *C*_max_ values from each rabbit, regardless of the time at which *C*_max_ was achieved); *T*_max_: time at which maximum plasma epinephrine concentration was achieved (mean ± SEM of individual *T*_max_ values in each rabbit); AUC_0−1 h_: area under the plasma concentration versus time curve (mean ± SEM of individual AUC values from each rabbit). * *n* = 5; ^†^ *p* < 0.05; ^††^ *T*_max_ is the time at which the highest peak epinephrine concentration occurred in each individual rabbit, regardless of the time since dosing. *T*_max_ is limited by experimental design because it is a discrete variable based on defined times of blood sampling.
